# Mycophenolate mofetil as maintenance therapy for proliferative lupus nephritis: a long-term observational prospective study

**DOI:** 10.1186/ar3184

**Published:** 2010-11-09

**Authors:** Katerina Laskari, Clio P Mavragani, Athanasios G Tzioufas, Haralampos M Moutsopoulos

**Affiliations:** 1 Department of Pathophysiology, School of Medicine, National and Kapodistrian University of Athens, Medical School, Mikras Asias Street 75, Goudi 11527, Athens, Greece; 2 Department of Experimental Physiology, School of Medicine, National and Kapodistrian University of Athens, Medical School, Mikras Asias Street 75, Goudi 11527, Athens, Greece

## Abstract

**Introduction:**

While the role of mycophenolate mofetil (MMF) in the management of lupus nephritis has been increasingly recognized, limited information is available regarding its efficacy and safety as a long-term maintenance treatment. The aim of the present study was to evaluate the efficacy and safety profile of MMF as maintenance therapy for proliferative lupus nephritis.

**Methods:**

Thirty-three consecutive patients with proliferative lupus nephritis received induction therapy with five to seven monthly intravenous (iv) pulses of cyclophosphamide (CYC) plus iv steroids followed by oral MMF 2 g/day as maintenance therapy for a median time of 29 months (range 9 to 71 months). Primary end points were the achievement of renal remission, complete renal remission, disease remission - renal and extrarenal -, the occurrence of renal relapse, chronic renal failure and death. Secondary end points were the extrarenal disease activity and drug adverse events. The clinical and laboratory parameters were compared during follow-up by means of nonparametric statistical tests. Time to event analysis was performed according to the Kaplan-Meier method.

**Results:**

A significant improvement of all renal parameters was observed at the end of the induction treatment and at the latest follow-up compared to baseline. The rate of patients achieving renal remission until the end of follow-up was 73%, whereas that of complete renal remission was 58%. The median survival times in the Kaplan-Meier analyses were 7 and 16 months, respectively. Remission was maintained in all but four (12%) patients who relapsed within 19 to 39 months after initial response. At the end of follow-up, 51% of the patients had reached disease remission. The median survival time of disease remission was 18 months. Extrarenal manifestations were well controlled in most of the patients. In one patient receiving MMF, extrarenal activity led to treatment discontinuation. Non life-threatening drug adverse events developed in 18 patients (58%) and included infections, amenorrhea, myelotoxicity, gastrointestinal complications, hypercholesterolemia, alopecia and drug intolerance. None of the patients developed chronic renal insufficiency or died from any cause.

**Conclusions:**

MMF appeared to be efficacious and safe as maintenance treatment for proliferative lupus nephritis.

## Introduction

Lupus nephritis, particularly the proliferative form, is among the most common and severe manifestations of systemic lupus erythematosus (SLE) leading to significant morbidity and mortality if left untreated [1]. Therapy aims to prevent evolution to end-stage renal disease and reduce mortality by early induction of remission and long-term prevention of recurrence. Intermittent intravenous (iv) pulses of cyclophosphamide (CYC) in combination with iv or oral steroids have been the standard of care for induction of remission, with long-term quarterly iv CYC pulses used as remission maintenance treatment [2,3]. However, the benefits of CYC have been limited by the significant drug-related toxicities including sustained amenorrhea as well as the possibility of no response or relapse in a substantial number of these patients [4-6]. In this context, alternative therapeutic modalities and the use of less toxic agents, such as mycophenolate mofetil (MMF) or azathioprine, have been sought [7,8].

MMF is a relatively new immunosuppressive agent initially used in solid organ transplantation with selective inhibitory effects on activated T and B lymphocytes. In recent years, MMF has been considered an important alternative agent for lupus nephritis refractory to other treatments and has also been studied as an induction therapy agent with promising results and mild toxicity [9-13]. However, recent prospective data have failed to demonstrate the superiority of MMF over iv CYC as an induction therapy [14]. Sequential regimens of short-term iv CYC followed by either MMF or azathioprine maintenance therapy have been shown to be efficacious and safe in reducing the long-term exposure to CYC, mainly in African-American or Hispanic patients [15]. The goal of the present study was to evaluate the efficacy and safety profile of MMF as maintenance therapy for proliferative lupus nephritis in a single center cohort of patients with proliferative lupus nephritis.

## Materials and methods

### Study design

Thirty-three consecutive patients with proliferative lupus nephritis class III (*n *= 20), IV (*n *= 7) or V with III/IV lesions (*n *= 6) according to the revised World Health Organization (WHO) classification [16] were recruited and prospectively followed up between 2001 and 2008. All patients received induction therapy with five to seven iv monthly pulses of CYC 1 g/m^2 ^(five pulses *n *= 5; six pulses *n *= 24; seven pulses *n *= 4) in association with iv pulses of 1 g methylprednisolone [2] followed by 2 g/day MMF according to a standardized protocol. The five patients who stopped induction therapy at five CYC pulses developed CYC-related drug side effects, while the four patients who received seven CYC pulses had active deteriorating renal disease. All patients fulfilled the American College of Rheumatology (ACR) classification criteria for SLE [17]. Exclusion criteria included non-adherence to the treatment protocol as well as irregular or lost follow-up (*n *= 3). Patients without renal biopsy or with a histological diagnosis of nephritis more than two months prior to treatment initiation were excluded from the study (*n *= 1).

All patients receiving CYC also received mesna (sodium-2-mercaptoethane sulfonate at three-fourths of the CYC dose) to prevent hemorrhagic cystitis and 16 mg of ondansetron to prevent nausea and vomiting with every CYC pulse. Oral methylprednisolone was given in all but one patient at the dose of 0.5 to 1 mg/kg/day for mild to moderate and severe extrarenal manifestations, respectively, with subsequent dose tapering based on extrarenal activity. As severe extrarenal manifestations were considered the involvement of the central nervous system, myocarditis, mesenteric vasculitis, hemolytic or aplastic anemia, thrombocytopenia < 50,000/mm^3^, leucopenia < 1,000/mm^3^, while as mild to moderate the presence of general symptoms, joint involvement, myalgias, acute rash, oral ulcers, serositis, myositis, pneumonitis, hepatosplenomegaly/increased liver enzymes, leucopenia > 1,000/mm^3 ^and thrombocytopenia > 50,000/mm^3^. No patient required the administration of iv steroids during the maintenance treatment.

Patients were followed up every month during induction therapy and every three months during maintenance treatment. During each visit the patients were evaluated by a complete physical examination as well as routine laboratory testing (blood count, biochemical tests, inflammatory markers, urine analysis and measurement of proteinuria in 24-hour urine collection). Moreover, drug side effects were recorded. The European Consensus Lupus Activity Measurement (ECLAM) score [18] was recorded at baseline, at the end of the induction treatment and at the latest follow-up. Renal-biopsy specimens were examined by light microscopy and immunofluorescence. Activity and chronicity indexes were estimated according to the scoring system of Austin *et al*. [19]. The presence of crescents (≥ 1/biopsy specimen), fibrinoid necrosis/karyorrhexis (≥ 1/biopsy specimen), interstitial fibrosis, tubular atrophy and glomerulosclerosis (≥ 1 lesion/biopsy specimen) was also recorded.

Informed consent was obtained from all patients. The design of the work has been approved by the hospital ethical committee and the study has been carried out in accordance with the Code of Ethics of the World Medical Association.

### End points and definitions

Primary end points were the achievement of renal remission, complete renal remission, disease remission, the occurrence of renal relapse, chronic renal failure and death. Secondary end points were the extrarenal disease activity and medication-related adverse events.

Renal remission was defined as the presence of all the criteria below in at least two measurements one month apart: a.) a decrease of ≥ 50% in proteinuria and proteinuria < 3 g/24 h; b.) absence of hematuria (red blood cells (RBCs) ≤ 5 hpf); c.) absence of pyuria (white blood cells (WBCs) ≤ 5 hpf), d.) absence of cellular casts (<1 hpf); and e.) stable (fluctuations within 10% of the initial value) glomerular filtration rate (GFR) if baseline serum creatinine < 2 mg/dl or improvement ≥ 30% if baseline serum creatinine ≥ 2.0 mg/dl. Renal relapse was defined as an: a.) increase of ≥ 50% in proteinuria and proteinuria > 1 g/24 h, and/or b.) hematuria (RBCs > 5 hpf), and/or c.) pyuria (WBCs > 5 hpf), and/or d.) cellular casts (≥ 1 hpf), and/or e.) a decrease of ≥ 30% in GFR in at least two measurements. Complete renal remission was considered if the patients presented with all the criteria below in at least two measurements one month apart: a.) proteinuria 24 h ≤ 500 mg, b.) RBCs ≤ 5 hpf, c.) WBCs ≤ 5 hpf, d.) absence of cellular casts (<1 hpf), and e.) GFR of ≥ 80 ml/minute/1.73^3^. Chronic renal failure was considered the sustained increase (for more than four months) in serum creatinine to at least twice the baseline value or the need for long-term dialysis, or renal transplantation. The above definitions were met according to the ACR response criteria for proliferative and membranous renal disease in SLE clinical trials [20]. The Modification of Diet and Renal Disease (MDRD) equation was used to determine GFR [21]. Only causes of renal abnormalities attributed to lupus nephritis were taken into consideration in the above definitions and other possible causes were always excluded. Disease remission was defined as the combination of complete renal remission and absence of extrarenal manifestations. Myelotoxicity was defined by the presence of cytopenia along with consistent features of myelosuppression on bone marrow biopsy. Amenorrhea was defined as the loss of three or more menstrual cycles, whereas sustained amenorrhea as the lack of menses for at least 12 months.

### Statistical analysis

Scaled and/or ordinal patient characteristics were compared during follow-up using the Wilcoxon test for paired observations and nominal parameters using the McNemar test. Time to event analysis was performed according to the Kaplan-Meier method. Results were considered significant when *P*-values were ≤ 0.05. Analysis was conducted in SPSS version 13. All *P*-values are two-tailed.

## Results

### Patient characteristics at baseline and during follow-up

The baseline patient characteristics are shown in Tables 1 and 2. The median duration of treatment was 29 months (range 9-71), while the median oral methylprednisolone dose until the end of follow-up was 7.6 (range 0-21.2) mg. Most patients had focal proliferative glomerulonephritis. Moderate activity and relatively low chronicity indexes were observed in renal biopsy (median 4 and 1, respectively). Adverse predictive factors such as proteinuria of nephrotic range, low GFR, crescents, fibrinoid necrosis, interstitial fibrosis and glomerulosclerosis were present in 36%, 58%, 31%, 27%, 53% and 53% of patients, respectively. Renal function deteriorated in 8 patients promptly after treatment initiation, while five of them presented with acute renal insufficiency. Hypertension was present in all but one of these patients at baseline.

**Table 1:** Patient characteristics

Age	30 (14 to 56)
Sex (M:F)	5:28
SLE duration (months)	10 (0 to 312)
Nephritis duration (months)	2 (0 to 56)
Active nephritis until treatment onset (months)^‡^	1 (0 to 15)
Renal biopsy	
WHO class III	20 (61)
IV	7 (21)
V with III/IV lesions	6 (18)
Activity index	4 (2 to 18)
Chronicity index	1 (0 to 6)
Crescents (cellular and/or fibrous)	10 (30)
Fibrinoid necrosis/Karyorrhexis	9 (27)
Interstitial fibrosis	17 (51)
Glomerulosclerosis	18 (54)
Tubular atrophy	22 (67)
Positive Anti-dsDNA antibodies	32 (97)
Positive Anti-Ro antibodies	16 (48)
Positive Anti-La antibodies	6 (18)
Positive Anti-Sm antibodies	4 (12)
Positive anti-U1RNP antibodies	8 (24)
Positive antiphospholipid antibodies	9/26 (35)
Low C3 at baseline (<70 mg/dl)	9/26 (35)
Low C4 at baseline (<10 mg/dl)	19/26 (73)

^‡ ^Proteinuria > 500 mg/24 h, and/or hematuria (> 5 hpf), and/or pyuria (> 5 hpf), and/or casts (> 1 hpf), and/or ≥ 30% decrease in GFR in at least two measurements. Values are expressed as either proportions or median (range).

M, male; F, female; SLE, systemic lupus erythematosus; WHO, World Health Organization; hpf, high power field; GFR, glomerular filtration rate.

**Table 2:** Renal parameters and outcome measures at baseline, at the end of the induction treatment and at the latest follow-up

Parameters	At baseline	At the end of the induction therapy	P*	At the latest follow-up	P*	P**
GFR (ml/minute/1.73 m^2^)	74 (21 to 156)	82 (27 to 184)	0.008	84 (33 to 156)	0.009	0.095
No of pts with low GFR (<80 ml/minute/1.73 m^2^)	19 (58)	15 (45)	0.049	9 (27)	0.001	0.070
GFR only in pts with low levels (ml/minute/1.73 m^2^)	62 (21 to 79)	58 (27 to 79)	0.393	63 (33 to 79)	0.374	0.059
Acute renal failure	5 (15)	0		0		
Proteinuria (g/24 h)	1.7 (0.2 to 10.6)	0.8 (0 to 7.1)	<0.001	0.3 (0 to 4.7)	0.001	0.155
No of pts with proteinuria (> 500 mg/24 h)	29 (88)	20 (61)	<0.001	14 (42)	0.004	0.109
> 3 g/24 h	12 (36)	6 (18)		5 (15)		
1 to 3 g/24 h	9 (27)	7 (21)		6 (18)		
Hematuria (> 5 hpf)	29 (88)	12 (36)	<0.001	11 (33)	<0.001	1.00
Pyuria (> 5 hpf)	18 (54)	11 (33)	<0.001	7 (21)	0.001	0.219
Cellular Casts (> 1 hpf)	10 (30)	2 (6)	<0.001	3 (9)	0.065	1.00
Active urine sediment	29 (88)	13 (39)	<0.001	11 (33)	<0.001	0.754
Hypertension (Systolic pressure > 140 or diastolic > 90 mmHg)	7 (21)	3 (9)	0.180	3 (9)	0.70	1.00
ECLAM score	8.2 (2.5 to 13.5)	2.7 (0 to 7)	<0.001	2.5 (0 to 7.5)	<0.001	0.165
Remission		15 (45)	-	24 (73)	-	<0.001
Complete remission		8 (24)	-	19 (58)	-	<0.001
Disease remission		4 (12)	-	17 (51)	-	<0.001
Renal relapse		0	-	4 (12)	-	0.125

* compared to baseline, ** compared to the end of the induction treatment. Values are expressed as either proportions or median (range).

GFR, glomerular filtration rate; ECLAM, European Consensus Lupus Activity Measurement; hpf, high power field.

**Figure 1:**
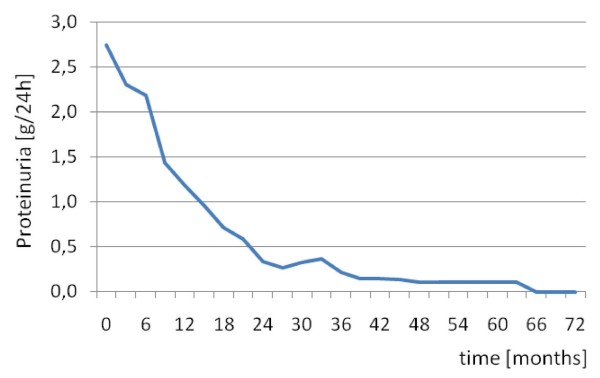
**Proteinuria values during follow-up**.

**Figure 2:**
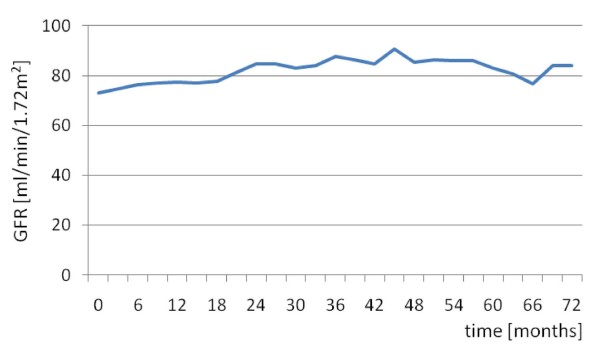
**GFR values during follow-up**.

Proteinuria resolved in 19 out of 29 (65%) patients within a median time of eight (range 1 to 30) months (Figure 1), whereas GFR normalized in 10 out of 19 (53%) patients within 10.5 (3 to 21) months (Figure 2). In six out of the eight patients with rapid renal function deterioration shortly after onset of treatment, GFR rates did not return to normal, however, at the end of follow-up, in all eight patients serum creatinine levels reached at least the baseline values. None of the patients received renal replacement therapy. Hematuria remitted in 21 out of 29 (72%) patients after a median (range) time of two (1 to 12) months and pyuria in 12 out of 18 (67%) patients within six (1 to 10) months.

A significant improvement of all renal parameters as well as ECLAM score was observed at the end of the induction treatment and at the latest follow-up compared to baseline (Table 2). A comparison between the end of the induction therapy and the end of follow-up revealed that certain parameters such as GFR and proteinuria were further improved during the maintenance treatment, however, with the most sharp changes being observed during the induction treatment with CYC (Table 2). Interestingly, when GFR values of the 19 patients with impaired renal function were compared during follow-up, a sharper, though non-significant, improvement was demonstrated during the maintenance but not during the induction treatment (Table 2).

### Outcome measures

#### Primary end points

##### Renal remission

**Figure 3:**
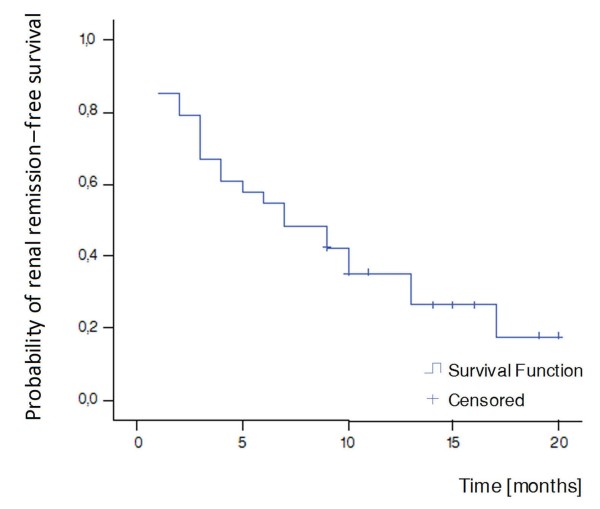
**Kaplan-Meier curve for renal remission**. Median survival time = seven months.

Fifteen out of 33 patients (45%) reached renal remission until the end of the induction treatment, whereas at the end of follow-up, the rates of patients achieving renal remission were 73% (Table 2). The median renal remission time in the Kaplan-Meier survival analysis was seven months (Figure 3).

##### Complete renal remission

**Figure 4:**
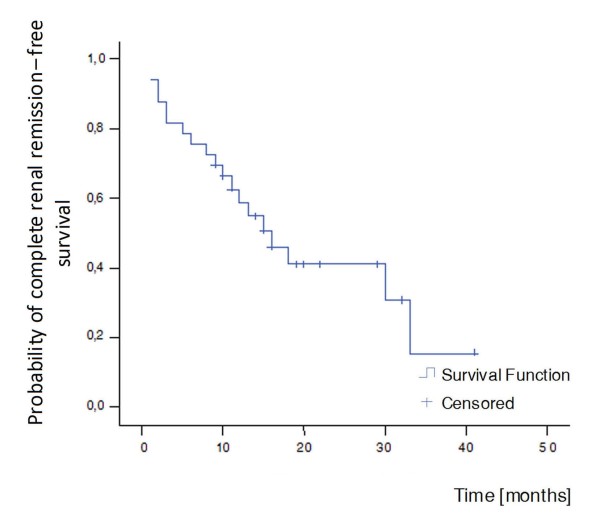
**Kaplan-Meier curve for complete renal remission**. Median survival time = 16 months.

Complete renal remission was achieved in 8 out of 33 patients (24%) at the end of the induction phase, while rates of complete remission of renal disease significantly increased to 58% (19 patients) until the latest follow-up (Table 2). The median survival time was 16 months (Figure 4).

##### Renal relapse

**Figure 5:**
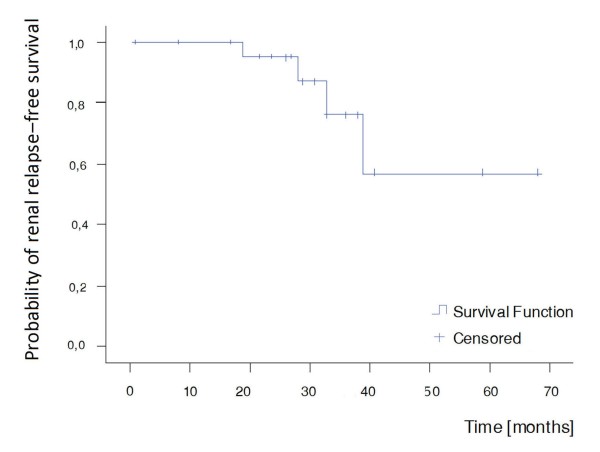
**Kaplan-Meier curve for renal relapse**. No median survival time.

Relapses occurred during the maintenance phase of therapy in 4 out of 24 patients in remission (12%) (Table 2, Figure 5). The time from remission to relapse ranged between 19 and 39 months, while the time from baseline to relapse ranged between 25 and 41 months. One patient was in both renal and complete renal remission for 19 months when she presented pyuria, a slight increase in proteinuria (600 mg in 24-hour urine collection), associated with fever, a vasculitic finger rash and an elevated erythrocyte sedimentation rate. Another patient being in renal and complete renal remission for 39 and 38 months, respectively, presented with hematuria. The third patient was in renal remission for 28 months, nevertheless, a low level of proteinuria (500 mg in 24-hour urine collection) persisted during follow-up. She relapsed with hematuria as well as an increase in proteinuria (1.1 g in 24-hour urine collection). Finally, the fourth patient was in renal remission for 33 months, and complete renal remission for 27 months, when proteinuria of 1.1 g in 24-hour urine collection as well as hypertension were observed. The mean ECLAM score of these four patients at the time of relapse was 5.1.

##### Disease remission

**Figure 6:**
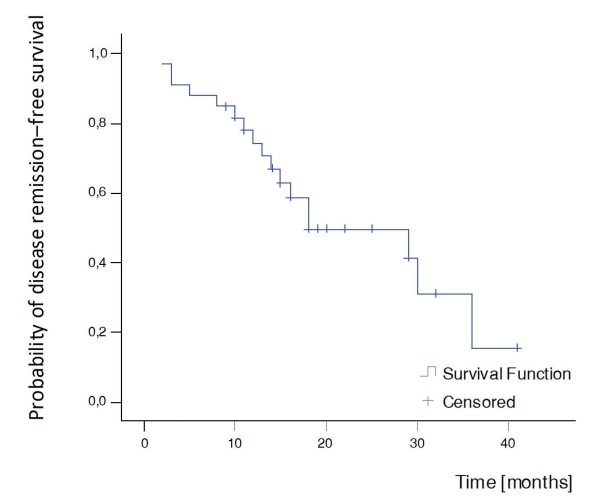
**Kaplan-Meier curve for disease remission**. Median survival time = 18 months.

Disease remission was observed in four patients (12%) at the end of the induction treatment. At the end of follow-up, 17 out of 33 (51%) patients had reached disease remission (Table 2). The median survival time in the Kaplan-Meier analysis was 18 months (Figure 6).

##### Chronic renal failure-death

None of the patients developed chronic renal insufficiency or died.

#### Secondary end points

##### Extrarenal manifestations

Extrarenal manifestations at baseline and during follow-up as well as the time to resolution of each symptom are shown in Table 3. The majority of the initial extrarenal manifestations resolved during the induction treatment; however, in some patients, the resolution of skin involvement, serositis, anemia and leucopenia was observed during the maintenance treatment (Table 3). Joint involvement, rash, constitutional symptoms and leucopenia were among the most frequently observed extrarenal manifestations during follow-up (Table 3). MMF was discontinued in one patient who developed shrinking lung syndrome 11 months after the onset of treatment.

**Table 3:** Extrarenal manifestations at baseline and during follow-up

Extrarenal manifestations	At baseline, pt no (%)	Pt no with baseline symptom resolution; Median months to symptom resolution (range)	New episodes, pt no (%)
General symptoms	18 (54)	18/18; 1 (1-2)	8 (24)
Rash	15 (45)	15/15; 2 (1-15)	9 (27)
Oral ulcers	4 (12)	4/4; 1.5 (1-5)	5 (15)
Arthralgias/arthritis	13 (39)	12/13; 1 (1-5)	10 (30)
Alopecia	4 (12)	4/4; 1.5 (1-2)	2 (6)
Myalgias	4 (12)	4/4; 1 (1-2)	2 (6)
Serositis	8 (24)	8/8; 1 (1-8)	2 (6)
Pneumonitis	2 (6)	2/2; 1.5 (1-2)	1 (3)
Thrombosis	2 (9) [1 PE, 2 DVTs]	2/2	0
CNS	2 (6) [brain infarcts]	1 both pts	1 (3) [seizures]
Myositis	0		1 (3)
Hepatosplenomegaly	5 (15)	5/5	1 (3)
Anemia (Hb < 12 g/dl for female and < 13.5 g/dl for male)	15 (45)	10/15; 4 (1-18)	5 (15)
Hemolytic anemia	8 (24)		2 (6)
Aplastic anemia	1 (3)		1 (3)
Leucopenia (<3.500/mm^3^)	13 (39)	11/13; 1 (1-14)	8 (24)
Thrombocytopenia (<100.000/mm^3^)	0		0
Increased liver enzymes	5 (15)	5/5; 1 (1-2)	2 (6)

PE, pulmonary embolism; DVT, deep vein thrombosis; CNS, central nervous system; Hb, hemoglobin.

##### Side effects

Eighteen out of the 33 patients experienced drug side effects (54%), (10 CYC-related and 12 MMF-related). Eight patients (24%) experienced severe infections during follow-up; three during the induction treatment (one fungal vaginitis, one systemic CMV infection, one sinusitis) and five during the maintenance treatment (four herpes zoster infections, one chlamydia-related myocarditis). Amenorrhea developed in 14% (4/28) of women and sustained amenorrhea in 4% (1/28). Three patients had CYC-related myelotoxicity. One patient developed a CYC-related infusion reaction (3%). No hemorrhagic cystitis was observed. Alopecia developed in one patient during MMF treatment (3%). Transient gastrointestinal complications were experienced by two patients during the maintenance treatment (6%) (one ulcerative gastritis, one gastrointestinal discomfort). Finally, hypercholesterolemia developed in four patients (12%) treated with MMF.

## Discussion

In the present study we aimed to investigate the safety and efficacy of MMF as sequential maintenance therapy for proliferative lupus nephritis following induction treatment with a short-course of iv CYC. Satisfactory response rates and acceptable tolerance profile were observed in most patients. Remission as well as complete renal remission occurred in a high percentage of patients, 73% and 58%, respectively, while relapse rates were low (12%). No severe complications such as chronic renal failure or death from any cause occurred. Moreover, a complete resolution of disease activity - renal and extrarenal - was evident in half of patients (51%).

Disease activity was suppressed in the majority of patients at the end of the induction treatment as evidenced by a significant improvement of all renal parameters. Furthermore, the majority of extrarenal manifestations resolved within the first months of treatment. Sequential therapy with MMF led to further improvement in renal disease outcome and maintained the initial response in the majority of patients. It is of note that renal remission occurred more frequently during induction therapy, whereas complete renal remission as well as disease remission were usually observed subsequently, during the maintenance treatment. At this point, we should emphasize the potential benefit from iv corticosteroid pulses in addition to CYC during the induction therapy. Given that previous evidence supports the beneficial role of iv corticosteroids over pulse CYC alone in the preservation of renal function in the long-term follow-up, we cannot exclude a long-term benefit from the use of iv methylprednisolone pulses in our patients [2].

While the majority of data regarding the therapeutic role of MMF is limited to lupus nephritis, its efficacy in lupus-related non-renal manifestations has not been widely studied. Limited evidence indicates that MMF may be effective in refractory hematological and dermatological manifestations; a reduced disease activity, as assessed by the SLEDAI (SLE disease activity index) and a significant reduction in the oral corticosteroid dose have also been described [22-25]. A recently published, multicenter, randomized clinical trial showed that MMF is a suitable alternative to CYC for the treatment of renal and non-renal disease manifestations in patients with biopsy-proven lupus nephritis [26]. In agreement with these observations, in our study, most of the baseline extrarenal manifestations resolved after treatment onset and new manifestations occurred relatively rarely.

In regard to toxicity, treatment with MMF after a short-course of CYC was shown to be safe and well tolerated in most of our patients. Infections, despite their severity, did not lead to life-threatening complications. On the other hand, gastrointestinal intolerance due to MMF was rare and reversible and the majority of women preserved ovarian function. This observation is in accord with the study of Ioannidis *et al*. suggesting that patients at high risk are those who exceed the total CYC dose of 12 g per body surface [4]. In regard to myelotoxicity, MMF was shown to be safe since overt bone marrow suppression was a complication of CYC and not MMF in our study, although serious myelotoxicity due to CYC has been previously reported to be rather uncommon [27]. Notably, no episodes of hemorrhagic cystitis occurred.

The decision on the maintenance treatment of proliferative lupus nephritis is an important issue in clinical practice. There is only one published randomized trial in the literature, the Contreras trial, providing data on patients treated with MMF maintenance therapy after a short course of iv CYC [15]. Our study supports the observations previously described with comparable renal remission and relapse rates. Moreover, a similar proportion of patients developed infections and nausea/vomiting, while the rates of women with sustained amenorrhea were comparable. In contrast to the study by Contreras *et al*., where one death (5%) due to severe infection and one episode of chronic renal failure (5%) occurred, in our study such outcomes were not observed. In a more recent retrospective study from Turkey including patients with proliferative but also membranous nephritis receiving the above sequential regimen, disease outcomes similar to ours were reported [28]. Nevertheless, diarrhea due to MMF was more frequently described in this cohort.

The comparison of MMF to the standard therapy of long-term iv CYC pulses as maintenance therapies for proliferative lupus nephritis has been studied in the trial by Contreras *et al*. MMF was shown to be superior over CYC both in terms of renal relapse and drug side effects (infections, amenorrhea, leucopenia). In line with these observations, we recently demonstrated a five-fold lower risk of sustained amenorrhea after a short duration treatment with CYC followed by MMF compared to long-term CYC administration (51% vs. 4%) [29]. Moreover, unpublished data on a historical cohort of 46 patients treated with long-term intermittent CYC pulses in our department, matched for age, sex and renal disease severity with the prospectively evaluated population, demonstrated fewer relapses during MMF maintenance treatment (12% vs. 22%), while remission rates between patients treated with CYC-MMF and the historical cohort were similar (73% vs. 70%). The existing literature on lupus nephritis treated with intermittent iv CYC pulses reveals similar concussions. Approximately 15 to 38% of patients did not respond to treatment with CYC in previous studies, while renal relapse occurred in 37% and 40% of patients in two studies and at a lower percentage (14%) in another one [2,5,6,14,15,30-32]. Taking into consideration that renal flares have been previously shown to be strong predictors of poor long-term renal outcome due to their potential for cumulative damage [33], the combination CYC-MMF emphasizes a potentially better long-term efficacy of MMF vs. CYC as maintenance therapy.

In addition to the end points studied by Contreras *et al*., in our study, we evaluated the achievement of complete renal remission. Interestingly, when compared to the historical cohort, MMF seemed to be superior over long-term intermittent CYC pulses (58% compared to 37% of the patients went into complete renal remission). The role of complete renal remission for renal and patient survival was investigated in the study by Chen *et al*. [34]. The renal survival at 10 years was 94% for complete remission, 45% for partial, and 19% for no remission, while the patient survival without end-stage renal disease at 10 years was 92% for complete, 43% for partial, and 13% for no remission. The above observation emphasizes the potential beneficial role of MMF for renal and patient survival in the long-term.

On the other hand, the already reported evidence on the use of azathioprine in the maintenance therapy of lupus nephritis has shown similar efficacy and toxicity to MMF [11,15,28]. Compared to the present results, sequential regimens of short-term CYC followed by azathioprine usually demonstrated slightly higher relapse rates; approximately 30% vs. 12% [7,11,15]. In our center, limited information is yet available regarding the use of azathioprine following induction therapy with iv CYC. More data are awaited in order to draw conclusions based on our population regarding the optimal substitute for CYC in the maintenance therapy of proliferative lupus nephritis.

The optimal treatment duration in patients with remitting proliferative lupus nephritis treated with MMF has not been clarified. Prospective controlled studies are awaited to address what is the optimal MMF dosage used for maintenance of remission and whether remission-maintenance therapy with MMF can be reduced or withdrawn safely. Preliminary, yet unpublished, data from our department support the possibility of gradual drug discontinuation in responders. Reducing MMF > 1.5 years after the achievement of remission and/or complete remission may warrant drug tapering without disease flaring.

Finally, our results should be interpreted in the context of potential limitations. The present study is an observational study and is limited by the absence of a randomized control group. Moreover, we should take into consideration that our cohort consisted of Caucasian patients. Since a better response to MMF has been previously demonstrated by non-Caucasian patients [12,14], our results might not have wide application. Nevertheless, the present study provides valuable information on the critical issue of maintenance treatment decision in lupus nephritis given the significant number of SLE patients studied, the long period of follow-up, the stringent definitions used for all the investigated parameters and clinical outcomes, and the opportunity to have detailed information on the patients' characteristics during a regular follow-up. Moreover, our results are strengthened by the comparison to an historic control group. Of course, larger controlled trials would ascertain our observations.

## Conclusions

In conclusion, the present study supports the efficacy and safety of MMF as maintenance treatment for proliferative lupus nephritis following an intensive induction therapy with a short-course of iv CYC. The benefit of MMF may translate to improved complete renal remission and relapse rates as well as reduction in CYC-associated toxicity, which predicts a better long-term disease outcome. Moreover, MMF appears to have beneficial effects in controlling the extrarenal manifestations of SLE.

## Abbreviations

ACR: American College of Rheumatology; CYC: cyclophosphamide; ECLAM SCORE: European Consensus Lupus Activity Measurement score; GFR: glomerular filtration rate; IV: intravenous; MDRD EQUATION: Modification of Diet and Renal Disease equation; MMF: mycophenolate mofetil; RBCS: red blood cells; SLE: systemic lupus erythematosus; SLEDAI: SLE disease activity index; WBCS: white blood cells; WHO: World Health Organization.

## Competing interests

The authors declare that they have no competing interests.

## Authors' contributions

KL participated in the design of the study, collected the data, performed the statistical analysis and interpretation of data, and drafted the article. CPM helped in drafting and revising the article and provided intellectual content of critical importance. AGT helped in revising the article and provided intellectual content of critical importance. HMM conceived of the study, participated in its design and coordination and provided intellectual content of critical importance. All authors read and approved the final manuscript.

## Authors' information

KL is Resident in Rheumatology at the Department of Pathophysiology, School of Medicine, University of Athens, Athens, Greece.

CPM is Lecturer at the Department of Experimental Physiology, School of Medicine, University of Athens, Athens, Greece.

AGT is Professor at the Department of Pathophysiology, School of Medicine, University of Athens, Athens, Greece.

HMM is Professor and Director at the Department of Pathophysiology, School of Medicine, University of Athens, Athens, Greece.

## References

[B1] Appel GB Cohen DJ Pirani CL Meltzer JI Estes D Long-term follow-up of patients with lupus nephritis: a study based on the classification of the World Health Organization Am J Med 1987 83 877 885 10.1016/0002-9343(87)90645-0 3674094

[B2] Illei GG Austin HA Crane M Collins L Gourley MF Yarboro CH Vaughan EM Kuroiwa T Danning CL Steinberg AD Klippel JH Balow JE Boumpas DT Combination therapy with pulse cyclophosphamide plus pulse methylprednisolone improves long-term renal outcome without adding toxicity in patients with lupus nephritis Ann Intern Med 2001 135 248 257 11511139 10.7326/0003-4819-135-4-200108210-00009

[B3] Austin HA Klippel JH Balow JE le Riche NG Steinberg AD Plotz PH Decker JL Therapy of lupus nephritis. Controlled trial of prednisone and cytotoxic drugs N Engl J Med 1986 314 614 619 10.1056/NEJM198603063141004 3511372

[B4] Ioannidis JPA Katsifis GE Tzioufas AG Moutsopoulos H Predictors of sustained amenorrhea from pulsed intravenous cyclophosphamide in premenopausal women with systemic lupus erythematosus J Rheumatol 2002 29 2129 2135 12375322

[B5] Dooley MA Hogan S Jennette C Falk R Cyclophosphamide therapy for lupus nephritis: poor renal survival in black Americans. Glomerular Disease Collaborative Network Kidney Int 1997 51 1188 1195 10.1038/ki.1997.162 9083285

[B6] Ioannidis JP Boki KA Katsorida ME Drosos AA Skopouli FN Boletis JN Moutsopoulos HM Remission, relapse, and re-remission of proliferative lupus nephritis treated with cyclophosphamide Kidney Int 2000 57 258 264 10.1046/j.1523-1755.2000.00832.x 10620207

[B7] Houssiau FA Vasconcelos C D'Cruz D Sebastiani GD Garrido Ed Ede R Danieli MG Abramovicz D Blockmans D Mathieu A Direskeneli H Galeazzi M Gül A Levy Y Petera P Popovic R Petrovic R Sinico RA Cattaneo R Font J Depresseux G Cosyns JP Cervera R Immunosuppressive therapy in lupus nephritis: the Euro-Lupus Nephritis triala randomized trial of low-dose versus high dose intravenous cyclophosphamide Arthritis Rheum 2002 46 2121 2131 10.1002/art.10461 12209517

[B8] Houssiau FA Vasconcelos C D'Cruz D Sebastiani GD de Ramon Garrido E Danieli MG Abramovicz D Blockmans D Cauli A Direskeneli H Galeazzi M Gül A Levy Y Petera P Popovic R Petrovic R Sinico RA Cattaneo R Font J Depresseux G Cosyns JP Cervera R The 10-year follow-up data of the Euro-Lupus Nephritis Trial comparing low-dose and high-dose intravenous cyclophosphamide Ann Rheum Dis 2010 69 61 64 10.1136/ard.2008.102533 19155235

[B9] Kapitsinou PP Boletis JN Skopouli FN Boki KA Moutsopoulos HM Lupus nephritis: treatment with mycophenolate Mofetil Rheumatology (Oxford) 2004 43 377 380 10.1093/rheumatology/keh012 14963204

[B10] Chan TM Li FK Tang CS Mok MY Li FK Hong Kong Nephrology Study Group Efficacy of mycophenolate mofetil in patients with diffuse proliferative lupus nephritis N Engl J Med 2000 343 1156 1162 10.1056/NEJM200010193431604 11036121

[B11] Chan TM Tse KC Tang CS Mok MY Li FK Hong Kong Nephrology Study Group Long-term study of mycophenolate mofetil as continuous induction and maintenance treatment for diffuse proliferative lupus nephritis J Am Soc Nephrol 2005 16 1076 84 10.1681/ASN.2004080686 15728784

[B12] Ginzler EM Dooley MA Aranow C Kim MY Buyon J Merrill JT Petri M Gilkeson GS Wallace DJ Weisman MH Appel GB Mycophenolate mofetil or intravenous cyclophosphamide for lupus nephritis N Engl J Med 2005 353 2219 2228 10.1056/NEJMoa043731 16306519

[B13] Cortés-Hernández J Torres-Salido MT Medrano AS Tarrés MV Ordi-Ros J Long-term outcomes--mycophenolate mofetil treatment for lupus nephritis with addition of tacrolimus for resistant cases Nephrol Dial Transplant 2010 25 3939 3948 10.1093/ndt/gfq322 20538787 20538787

[B14] Appel GB Contreras G Dooley MA Ginzler EM Isenberg D Jayne D Li LS Mysler E Sánchez-Guerrero J Solomons N Wofsy D Aspreva Lupus Management Study Group Mycophenolate mofetil versus cyclophosphamide for induction treatment of lupus nephritis J Am Soc Nephrol 2009 20 1103 1112 10.1681/ASN.2008101028 19369404 PMC2678035

[B15] Contreras G Pardo V Leclercq B Lenz O Tozman E O'Nan P Roth D Sequential therapies for proliferative lupus nephritis N Engl J Med 2004 350 971 980 10.1056/NEJMoa031855 14999109

[B16] Weening JJ D'Agati VD Schwartz MM Seshan SV Alpers CE Appel GB Balow JE Bruijn JA Cook T Ferrario F Fogo AB Ginzler EM Hebert L Hill G Hill P Jennette JC Kong NC Lesavre P Lockshin M Looi LM Makino H Moura LA Nagata M The classification of glomerulonephritis in systemic lupus erythematosus revisited J Am Soc Nephrol 2004 15 241 250 10.1097/01.ASN.0000108969.21691.5D 14747370

[B17] Tan EM Cohen AS Fries JF Masi AT McShane DJ Rothfield NF Schaller JG Talal N Winchester RJ The 1982 revised criteria for the classification of systemic lupus erythematosus Arthritis Rheum 1982 25 1271 1277 10.1002/art.1780251101 7138600

[B18] Vitali C Bencivelli W Isenberg DA Smolen JS Snaith ML Sciuto M Neri R Bombardieri S Disease activity in systemic lupus erythematosus: report of the consensus study group of the European workshop for rheumatology research. II. Identification of the variables indicative of disease activity and their use in the development of an activity score Clin Exp Rheumatol 1992 10 541 547 1458710

[B19] Austin HA Muenz LR Joyce KM Antonovych TT Balow JE Diffuse proliferative lupus nephritis: identification of specific pathologic features affecting renal outcome Kidney Int 1984 25 689 695 10.1038/ki.1984.75 6482173

[B20] Renal Disease Subcommittee of the American College of Rheumatology Ad Hoc Committee on Systemic Lupus Erythematosus Response Criteria The American College of Rheumatology response criteria for proliferative and membranous renal disease in systemic lupus erythematosus clinical trials Arthritis Rheum 2006 54 421 432 10.1002/art.21625 16453282

[B21] Kasitanon N Fine DM Haas M Magder LS Petri M Estimating renal function in lupus nephritis: comparison of the Modification of Diet in Renal Disease and Cockcroft Gault equations Lupus 2007 16 887 895 10.1177/0961203307084167 17971362

[B22] Kreuter A Tomi NS Weiner SM Huger M Altmeyer P Gambichler T Mycophenolate sodium for subacute cutaneous lupus erythematosus resistant to standard therapy Br J Dermatol 2007 156 1321 1327 10.1111/j.1365-2133.2007.07826.x 17408395

[B23] Karim MY Alba P Cuadrado MJ Abbs IC D'Cruz DP Khamashta MA Hughes GR Mycophenolate mofetil for systemic lupus erythematosus refractory to other immunosuppressive agents Rheumatology (Oxford) 2002 41 876 882 10.1093/rheumatology/41.8.876 12154204

[B24] Vasoo S Thumboo J Fong KY Refractory immune thrombocytopenia in systemic lupus erythematosus: response to mycophenolate mofetil Lupus 2003 12 630 632 10.1191/0961203303lu417cr 12945723

[B25] Mak A Mok CC Mycophenolate mofetil for refractory haemolytic anemia in systemic lupus erythematosus Lupus 2005 14 856 858 10.1191/0961203305lu2163cr 16302683

[B26] Ginzler EM Wofsy D Isenberg D Gordon C Lisk L Dooley MA ALMS Group Nonrenal disease activity following mycophenolate mofetil or intravenous cyclophosphamide as induction treatment for lupus nephritis: findings in a multicenterprospectiverandomizedopen-labelparallel-group clinical trial Arthritis Rheum 2010 62 211 221 10.1002/art.25052 20039429

[B27] Katsifis GE Tzioufas AG Vlachoyiannopoulos PG Voulgarelis M Moutsopoulos HM Ioannidis JP Risk of myelotoxicity with intravenous cyclophosphamide in patients with systemic lupus erythematosus Rheumatology (Oxford) 2002 41 780 786 10.1093/rheumatology/41.7.780 12096228

[B28] Sahin GM Sahin S Kiziltas S Masatlioglu S Oguz F Ergin H Mycophenolate mofetil versus azathioprine in the maintenance therapy of lupus nephritis Ren Fail 2008 30 865 869 10.1080/08860220802353843 18925525

[B29] Laskari K Zintzaras E Tzioufas AG Ovarian function is preserved in women with severe systemic lupus erythematosus after a 6-month course of cyclophosphamide followed by mycophenolate mofetil Clin Exp Rheumatol 2010 28 83 86 20346244

[B30] Mok CC Ho CT Siu YP Chan KW Kwan TH Lau CS Wong RW Au TC Treatment of diffuse proliferative lupus glomerulonephritis: a comparison of two cyclophosphamide-containing regimens Am J Kidney Dis 2001 38 256 264 10.1053/ajkd.2001.26084 11479150

[B31] Gourley MF Austin HA Scott D Yarboro CH Vaughan EM Muir J Boumpas DT Klippel JH Balow JE Steinberg AD Methylprednisolone and cyclophosphamidealone or in combinationin patients with lupus nephritis. A randomizedcontrolled trial Ann Intern Med 1996 125 549 557 8815753 10.7326/0003-4819-125-7-199610010-00003

[B32] Boumpas DT Austin HA Vaughn EM Klippel JH Klippel JH Steinberg AD Yarboro CH Balow JE Controlled trial of pulse methylprednisolone versus two regimens of pulse cyclophosphamide in severe lupus nephritis Lancet 1992 340 741 745 10.1016/0140-6736(92)92292-N 1356175

[B33] Moroni G Quaglini S Maccario M Banfi G Ponticelli C "Nephritic flares" are predictors of bad long-term renal outcome in lupus nephritis Kidney Int 1996 50 2047 2053 10.1038/ki.1996.528 8943489

[B34] Chen YE Korbet SM Katz RS Schwartz MM Lewis EJ for the Collaborative Study Group Value of a complete or partial remission in severe lupus nephritis Clin J Am Soc Nephrol 2008 3 46 53 10.2215/CJN.03280807 18003764 PMC2390978

